# Short-term pelvic fracture outcomes in adolescents differ from children and adults in the National Trauma Data Bank

**DOI:** 10.1007/s11832-015-0634-3

**Published:** 2015-02-04

**Authors:** Meir Marmor, Joshua Elson, Christopher Mikhail, Saam Morshed, Amir Matityahu

**Affiliations:** 1 Orthopaedic Trauma Institute (OTI), San Francisco General Hospital, University of California, San Francisco (UCSF), 2550 23rd Street, Building 9, 2nd Floor, San Francisco, CA 94110 USA; 2 Medical School for International Health, Ben-Gurion University of the Negev, Beer Sheba, Israel; 3 Rush University, 600 S Paulina St., Chicago, IL 60612 USA

**Keywords:** Pelvic fractures, Adolescents, Children, Pediatric, Outcomes, National Trauma Data Bank

## Abstract

**Background:**

Pediatric pelvic fractures are associated with high-energy trauma and injury to other systems, leading to an increased incidence of complication and mortality. Previous studies analyzed the pediatric population as a whole, including both children and adolescents. The purpose of this study was to examine whether adolescents with pelvic fracture have different complication and mortality rates compared to younger children and adults.

**Methods:**

Using the National Trauma Data Bank, 37,784 patients below the age of 55 years with pelvic fractures were identified and divided into children (age <13 years), adolescents (age 13–17 years), and adults (age >17 years). Descriptive statistics and bivariate and multivariate analyses were performed.

**Results:**

Children had an increased odds of death [odds ratio (OR) 2.29, 95 % confidence interval (CI) 1.96–2.67] and complications (OR 1.36, 95 % CI 1.20–1.55), whereas adolescents had a decrease in odds of death (OR 0.89, 95 % CI 0.74–1.06) and complications (OR 0.70, 95 % CI 0.61–0.81) compared to the adult population.

**Conclusions:**

Adolescents with pelvic fractures exhibit a different physiologic response to the children and adult populations. This emphasizes the need to distinguish these subpopulations in future epidemiological research and treatment planning.

## Background

Pelvic fractures comprise <0.2 % of all pediatric fractures but constitute up to 5 % of admissions to level I pediatric trauma centers [1]. Like adults, pediatric pelvic fractures are associated with high-energy trauma and injury to other systems, leading to an increased incidence of complication and mortality [2–4]. Due to the pliable nature of the child’s skeleton, severe soft tissue trauma may occur without producing skeletal injury [4, 5]. Thus, a child presenting with a pelvic fracture should be suspected of having a multiorgan injury with an increased potential for injury, complications, and death [6, 7]. During the transition period between childhood and adulthood, known as adolescence, children gain size and strength, making their body increasingly resistant to extrinsic injury. However, previous studies on pediatric pelvic fractures analyzed the pediatric population as a single group of both children and adolescents [2, 3, 7–11].

Biological, psychological, social, and environmental changes influence the onset and termination of adolescence [12]. Physiologic changes that occur during adolescence, such as increase in muscle size and bone mass, doubling in heart size and lung vital capacity, and rise in blood pressure, blood volume, and hematocrit (particularly in boys), may lead to a theoretical protective effect against trauma [12]. Adolescents also retain the ability to rapidly heal and recover. Similar to growing children, adolescents have an osteoblast:osteoclast activity ratio >1, which increases their capacity to heal fractures. These physiological advantages, together with the increase in distance between internal organs, weight to surface area ratio, and cardiovascular reserve [4], may make adolescents particularly resilient to traumatic injury.

The purpose of this study is to examine whether adolescents with pelvic fracture have different complication and mortality rates compared to younger children and adults.

## Methods

We identified the study population by means of the National Trauma Data Bank (NTDB version 7.1). This NTDB version contained over 2.7 million cases from over 900 US trauma centers between the years 2002 and 2006 [13]. The data were imported and merged into a single dataset from the 13 NTDB files using SAS^®^ version 9.2 (SAS Institute, Cary, NC).

The initial NTDB population consisted of over 2.7 million entries. For the purpose of our study, all burn or penetrating injuries were excluded, which reduced the group to 1.7 million cases. If a patient’s multiple ICD-9 diagnosis codes contained at least one of the following codes, the patient was considered to have a pelvic fracture: 808.2, 808.3, 808.4, 808.41, 808.42, 808.43, 808.49, 808.5, 808.51, 808.52, 808.53, 808.59, 808.8, 808.9. Acetabular fractures were excluded from the study. Those entries without a pelvic fracture were removed, giving a total of 54,459 cases of pelvic fractures. Finally, all adults aged 55 years and older were removed from the study, yielding a final study population of 37,784.

This final study population was subdivided into our three groups of interest: children (younger than 13 years old), adolescents (aged 13–17 years), and adults (aged 18–54 years). The children and adolescents populations together were identified as the pediatric population. Open fractures constituted 3.4 % of all fractures. Each subgroup was assessed for their odds of death and a severe complication. The main outcomes of interest were mortality and a severe complication. Severe complication was defined as having at least one of the following complications recorded: renal failure, pneumonia, bacteremia, acute respiratory distress syndrome (ARDS), deep vein thrombosis (DVT), or a pulmonary embolism. Prehospital risk factors such as sex, race, age, arrival in shock (systolic blood pressure <90 mmHg), Injury Severity Score (ISS), head injury, and mechanism of injury were also analyzed to determine their association with the main outcomes.

### Statistical analysis

All statistical analyses were conducted using SAS^®^. Descriptive statistics were performed on the entire study population (*n* = 37,784).

To determine associations between risk factors and the main outcomes of interest, bivariate analysis was conducted between each prehospital risk factor and two main outcomes (mortality and severe complication). Risk factors that were not already dichotomous were transformed into two mutually exclusive categories. For example, ISS became ≥25 and <25. For this and subsequent assessment, open fractures (only 4.2 % of pelvic fractures and highly variable in presentation and complication profile) and unknown mechanisms of injury were excluded, yielding a study group of 24,684.

A subpopulation of severe pelvic injuries was also created to determine if any pediatric subgroup has better outcomes. For this population, an Abbreviated Injury Scale (AIS) score of <3, signifying minor and moderate injuries, and all patients with lower extremity fractures besides pelvic fractures were excluded. This created a study subgroup of 2,487 patients with isolated severe pelvic fractures.

For both the main study population and the subgroup of severe pelvic injury, prehospital risk factors were determined to be significant using the Mantel–Haenszel test. This method produced crude odds ratios (ORs) and 95 % confidence intervals (CIs), and those variables of significance were included for the multivariate analysis.

Logistic regression analyses including those variables of significance were performed to determine the association between the prehospital risk factors and the two main outcomes. Each model included a specific age group (children, adolescent, and adult) as compared to all other ages in order to assess the importance of age and outcome after sustaining a pelvic fracture. The Hosmer–Lemeshow goodness-of-fit test was performed on each model to determine whether or not the observed event rates matched the expected event rates.

## Results

Our study’s overall incidence rate of pelvic fractures was 26 cases per 10,000 trauma admissions per year for adults, 4 cases per 10,000 trauma admissions per year for adolescents, and 5 cases per 10,000 trauma admissions per year for children. Severe pelvic fractures had an incidence rate of 3.5 cases per 10,000 per year for adults, 0.47 cases per 10,000 per year for adolescents, and 0.63 cases per 10,000 per year for children.

### Descriptive data for the whole pelvic fracture population

There were 37,784 individuals that matched the preliminary inclusion and exclusion criteria. Descriptive statistics of demographics, injury mechanism, injury severity, treatment, and complications are presented in Table 1. After sustaining a pelvic fracture, survival was 91.4 % for the total population. When separated by age group, adolescents appeared to have the best survival statistics (93.2 %), while children had the worst (89.8 %). At least one severe complication occurred in 14.9 % of the total population. Adolescents had the lowest percentage of severe complication (11.4 %), children had a slightly higher percentage (13.0 %), and adults had the highest percentage (15.7 %).

**Table 1:** Frequency distribution of characteristics for the entire population of pelvic fractures, *n* = 37,784

Variable	Age group			Total, *n* (%)
	Children (<13 years), *n* (%), *N* = 5,325	Adolescents (13–17 years), *n* (%), *N* = 4,052	Adults (18–54 years), *n* (%), *N* = 28,407	
Gender				
Male	2,349 (44.8)	1,888 (46.7)	17,393 (61.3)	21,630 (57.4)
Female	2,897 (55.2)	2,157 (53.3)	10,983 (38.7)	16,037 (42.6)
Race				
Caucasian	3,570 (72.3)	2,745 (73.4)	18,301 (69.9)	24,616 (70.7)
African American	474 (9.6)	341 (9.1)	2,717 (10.4)	3,532 (10.1)
Asian/Pacific Islander	73 (1.5)	54 (1.4)	504 (1.9)	631 (1.8)
Hispanic	490 (9.9)	394 (10.6)	3002 (11.5)	3,886 (11.2)
Native American	31 (0.6)	31 (0.9)	201 (0.8)	263 (0.8)
Other	299 (6.1)	173 (4.6)	1,440 (5.5)	1,912 (5.4)
Survival				
Survived	4,766 (89.8)	3,753 (93.2)	25,853 (91.5)	34,372 (91.4)
Died	542 (10.2)	273 (6.8)	2,404 (8.5)	3,219 (8.6)
Severe complication				
Yes	693 (13.0)	462 (11.4)	4,465 (15.7)	5,620 (14.9)
No	4,632 (87.0)	3,590 (88.6)	23,942 (84.3)	32,164 (85.1)
Fracture type				
Open	183 (3.4)	96 (2.4)	1,316 (4.6)	1,595 (4.2)
Closed	5,142 (96.6)	3,956 (97.6)	27,078 (95.4)	36,176 (95.8)
Fracture location				
Ilium	703 (13.7)	622 (15.7)	3,797 (13.9)	5,122 (14.1)
Ischium	162 (3.1)	153 (3.9)	1,151 (4.2)	1,466 (4.0)
Pubis	2,615 (42.0)	1,632 (41.2)	11,281 (41.4)	15,078 (41.5)
Multiple	330 (6.4)	220 (5.5)	1,799 (6.6)	2,349 (6.5)
Unspecified	673 (13.1)	342 (8.6)	2,328 (8.6)	3,343 (9.2)
Other	1,120 (21.7)	996 (25.1)	6,878 (25.3)	8,994 (24.7)
Systolic blood pressure (mmHg)				
<90	878 (16.5)	524 (12.9)	4,615 (16.3)	6,017 (15.9)
90–139	2,869 (53.9)	2,680 (66.2)	16,704 (58.8)	22,253 (58.9)
140+	1,578 (29.6)	848 (20.9)	7,088 (24.9)	9,514 (25.2)
MOI				
Motor vehicle	1,329 (24.9)	2,224 (54.9)	11,520 (40.6)	15,073 (39.9)
Motorcycle	119 (2.2)	91 (2.3)	2,674 (9.4)	2,884 (7.6)
Pedestrian	170 (3.2)	102 (2.5)	566 (1.9)	838 (2.2)
Crush	93 (1.8)	31 (0.7)	732 (2.6)	856 (2.3)
High fall	592 (11.1)	108 (2.6)	2,680 (9.5)	3,380 (9.0)
Low fall	1,216 (22.8)	63 (1.6)	1,393 (4.9)	2,672 (7.1)
Unknown	1,806 (34.0)	1,433 (35.4)	8,842 (31.1)	12,081 (31.9)
AIS of pelvis				
None	3,323 (62.4)	2,660 (65.6)	19,331 (68.1)	25,314 (67.0)
1	9 (0.2)	11 (0.3)	96 (0.3)	116 (0.3)
2	1,313 (24.6)	869 (21.5)	5,153 (18.2)	7,335 (19.4)
3	610 (11.5)	450 (11.1)	3,129 (11.0)	4,189 (11.1)
4	53 (1.0)	45 (1.1)	522 (1.8)	620 (1.6)
5	17 (0.3)	17 (0.4)	176 (0.6)	210 (0.6)
6	0 (0.0)	0 (0.0)	0 (0.0)	0 (0.0)
ISS				
<25	4,338 (81.5)	2,816 (69.5)	19,721 (69.4)	26,875 (71.1)
≥25	987 (18.5)	1,236 (30.5)	8,686 (30.6)	10,909 (28.9)
Pneumonia				
Yes	102 (1.9)	125 (3.1)	1,232 (4.3)	1,459 (3.9)
No	5,223 (98.1)	3,927 (96.9)	27,175 (95.7)	36,325 (96.1)
Acute respiratory stress syndrome				
Yes	61 (1.1)	55 (1.4)	573 (2.0)	689 (1.8)
No	5,264 (98.9)	3,997 (98.6)	27,834 (98.0)	37,095 (98.2)
Deep vein thrombosis				
Yes	27 (0.5)	21 (0.5)	503 (1.8)	551 (1.5)
No	5,298 (99.5)	4,031 (99.5)	27,904 (98.2)	37,233 (98.5)
Bacteremia				
Yes	10 (0.2)	26 (0.6)	196 (0.7)	232 (0.6)
No	5,315 (99.8)	4,026 (99.4)	28,211 (99.3)	37,501 (99.4)
Renal failure				
Yes	32 (0.6)	15 (0.4)	225 (0.8)	272 (0.7)
No	5,293 (99.4)	4,037 (99.6)	28,182 (99.2)	37,512 (99.3)
Pulmonary embolism				
Yes	15 (0.3)	5 (0.1)	160 (0.6)	180 (0.4)
No	5,310 (99.7)	4,047 (99.9)	28,247 (99.4)	37,604 (99.6)
ICU stay				
<1 week	4,890 (91.8)	3,545 (87.5)	24,086 (84.8)	32,521 (86.1)
1 week	435 (8.2)	507 (12.5)	4,321 (15.2)	5,263 (13.9)
Hospital stay				
<1 week	3,727 (70.0)	2,406 (59.4)	13,778 (48.5)	19,911 (52.7)
1 week	1,598 (30.0)	1,646 (40.6)	14,629 (51.5)	17,873 (47.3)
Major procedure				
CRIF	1 (0.02)	2 (0.05)	20 (0.07)	23 (0.08)
ORIF	245 (4.62)	395 (9.71)	4,691 (16.47)	5,331 (14.1)
Closed dislocation reduction	7 (0.13)	19 (0.47)	117 (0.41)	143 (0.39)
Open dislocation reduction	12 (0.23)	15 (0.37)	127 (0.45)	154 (0.43)
No major procedure	5,060 (95.0)	3,621 (89.4)	23,452 (82.6)	32,133 (85.0)

*MOI* mechanism of injury, *AIS* Abbreviated Injury Scale, *ISS* Injury Severity Score, *ICU* intensive care unit, *CRIF* closed reduction internal fixation, *ORIF* open reduction internal fixation

Most of the pelvic fractures were closed (95.8 %). Pubic rami fractures consistently had the highest prevalence across all age groups (41.5 %). The most common known mechanism of injury was motor vehicle accident (39.9 %), followed by high-energy fall (9.0 %) and motorcycle accident (7.6 %). Most patients had an ISS <25 (71.1 %). When divided by age, more children had an ISS <25 (81.5 %) than adolescents (69.5 %) or adults (69.4 %). Adolescents also had less reports of hypovolemic shock on arrival (systolic pressures below 90 mmHg) (12.9 %) than children (16.5 %) and adults (16.3 %) who had comparable numbers.

### Bivariate analysis for the whole pelvic fracture population

For this assessment, open fractures and unknown mechanisms of injury were excluded, yielding a study group of 24,684. The results of the bivariate analysis (reporting crude ORs and 95 % CIs) for this population are depicted in Table 2. As compared to the rest of the population, children had a small increase in odds of death (OR 1.27, 95 % CI 1.11–1.44), but a slight decrease in odds of severe complication (OR 0.83, 95 % CI 0.75–0.93). Adolescents had decreases in both odds of death (OR 0.85, 95 % CI 0.72–1.00) and severe complication (OR 0.78, 95 % CI 0.69–0.89). Adults had a slight decrease in odds of death (OR 0.92, 95 % CI 0.82–1.02), but a small increase in odds of severe complication (OR 1.27, 95 % CI 1.16–1.39). Individuals suffering from hypovolemic shock had substantially increased odds of death (OR 5.89, 95 % CI 5.32–6.51) and severe complication (OR 3.62, 95 % CI 3.33–3.93). Sustaining a head injury also increased the odds of death (OR 2.96, 95 % CI 2.59–3.39) and complication (OR 3.23, 95 % CI 2.90–3.61). An ISS ≥25 greatly increased the odds of death (OR 11.1, 95 % CI 9.92–12.5) and complication (OR 9.19, 95 % CI 8.48–9.97). Motor vehicle accidents caused the largest increase in odds of death (OR 1.46, 95 % CI 1.32–1.62) and severe complication (OR 1.53, 95 % CI 1.42–1.65).

**Table 2:** Bivariate analysis for the entire population of closed pelvic fractures comparing predictors upon arrival to the emergency department with the outcomes death and severe complication (odds ratios and 95 % confidence intervals), *n* = 24,684

Predictors	Outcome	
	Death	Severe complication^a^
Male	1.24 (1.13–1.37)	1.41 (1.31–1.52)
Not Caucasian	1.14 (1.04–1.26)	1.00 (0.92–1.08)
Pediatric (<18 years)	1.09 (0.98–1.21)	0.79 (0.72–0.86)
Children (<13 years)	1.27 (1.11–1.44)	0.83 (0.75–0.93)
Adolescent (13–17 years)	0.85 (0.72–1.00)	0.78 (0.69–0.89)
Adults (18–54 years)	0.92 (0.82–1.02)	1.27 (1.16–1.39)
Hypovolemic shock	5.89 (5.32–6.51)	3.62 (3.33–3.93)
Head injury	2.96 (2.59–3.39)	3.23 (2.90–3.61)
ISS ≥25	11.1 (9.92–12.5)	9.19 (8.48–9.97)
Motor vehicle accident	1.46 (1.32–1.62)	1.53 (1.42–1.65)
Motorcycle accident	1.34 (1.16–1.54)	1.34 (1.20–1.49)
Fall from height	0.61 (0.52–0.72)	0.61 (0.54–0.69)
Low-energy fall	0.41 (0.33–0.51)	0.36 (0.30–0.43)
Crush	0.43 (0.29–0.63)	0.66 (0.52–0.84)
Pedestrian versus auto	1.40 (1.11–1.77)	1.13 (0.93–1.38)

*ISS* Injury Severity Score

^a^Severe complication is defined as having one or more of the following during hospital course: pneumonia, bacteremia, deep vein thrombosis, pulmonary embolism, renal failure, acute respiratory distress syndrome, or death

### Multivariate analysis for the whole pelvic fracture population

Variables that were found to be significant in the bivariate analysis were included in the final multiple logistic regression models for death and severe complication as outcomes. Adjusted ORs and 95 % CIs are presented in Table 3. When controlling for mechanism of injury and other prehospital conditions, children had more than twice the odds of death (OR 2.29, 95 % CI 1.96–2.67) and increased odds of severe complication (OR 1.36, 95 % CI 1.20–1.55) relative to the adult group after sustaining a pelvic fracture. Adolescents, on the other hand, had a slight decrease in odds of death (OR 0.89, 95 % CI 0.74–1.06) and lower odds of severe complication (OR 0.70, 95 % CI 0.61–0.81) relative to the adult group after sustaining a pelvic fracture.

**Table 3:** Multivariate logistic regression analysis for the entire population of closed pelvic fractures using predictors upon arrival to the emergency department with the outcomes death and severe complication (odds ratios and 95 % confidence intervals)

Model predictors	Outcome	
	Death, *n* = 24,684	Severe complication^a^, *n* = 22,846
Children (<13 years)	**2**.**29** (**1**.**96**–**2**.**67**)	**1**.**36** (**1**.**20**–**1**.**55**)
Adolescents (13–17 years)	0.89 (0.74–1.06)	**0**.**70** (**0**.**61**–**0**.**81**)
Adults (18–54 years)	Referent	Referent
Male	**1**.**19** (**1**.**07**–**1**.**33**)	**1**.**38** (**1**.**26**–**1**.**50**)
Not Caucasian	1.00 (0.90–1.12)	–
Hypovolemic shock	**4**.**52** (**4**.**05**–**5**.**05**)	**2**.**81** (**2**.**56**–**3**.**08**)
Head injury	**1**.**60** (**1**.**37**–**1**.**86**)	**1**.**70** (**1**.**50**–**1**.**92**)
ISS ≥25	**9**.**15** (**8**.**07**–**10**.**4**)	**7**.**41** (**6**.**79**–**8**.**09**)
Motor vehicle accident	3.98 (0.39–40.2)	1.13 (0.92–1.41)
Motorcycle accident	4.61 (0.46–46.8)	1.18 (0.92–1.51)
Fall from height	3.27 (0.32–33.1)	0.80 (0.63–1.03)
Low-energy fall	3.18 (0.32–31.7)	0.77 (0.58–1.02)
Pedestrian versus auto	4.50 (0.45–44.8)	–
Crush	2.42 (0.23–25.3)	0.95 (0.69–1.34)
Hosmer–Lemeshow^b^	0.0069	0.17

Bold values signify 95 % confidence intervals that are completely greater than 1, indicating a significant positive association

*ISS* Injury Severity Score

^a^Severe complication is defined as having one or more of the following during hospital course: pneumonia, bacteremia, deep vein thrombosis, pulmonary embolism, renal failure, acute respiratory distress syndrome, or death

^b^Hosmer–Lemeshow *p*-value given for the model’s goodness of fit instead of odds ratio and 95 % confidence interval

### Descriptive data for the severe pelvic fracture subpopulation

A subgroup analysis was performed for patients determined to have a severe, isolated, closed pelvic injury (AIS ≥3). Table 4 demonstrates the descriptive statistics for this subpopulation (*n* = 2,487). Gender and race had no significant impact on outcomes compared to the whole population. Adolescents (*n* = 255) still appeared to have a lower incidence of mortality (3.1 %) and lower incidence of a severe complication (8.2 %) compared to adults (*n* = 1,886) and children (*n* = 346). Adolescents contributed the most to motor vehicle accident (89.4 %), which was the most common mechanism of injury in the severe pelvic fracture group. Similar to the whole population, the severe pelvic injury group demonstrated more children with an ISS <25 (82.1 %). For cases of hypovolemic shock on arrival, adolescents had the fewest reports of systolic pressures below 90 mmHg (6.7 %), while children (15.9 %) had the largest contribution. Children had the lowest incidence of hospital stay and intensive care unit (ICU) admission, while adolescents and adults were relatively comparable.

**Table 4:** Frequency distribution of characteristics for individuals with isolated, closed, severe pelvic fractures (AIS≥3), *n* = 2,487

Variable	Age group			Total, *n* (%)
	Children (<13 years), *n* (%), *N* = 346	Adolescents (13–17 years), *n* (%), *N* = 255	Adults (18–54 years), *n* (%), *N* = 1,886	
Gender				
Male	127 (38.7)	92 (36.2)	1,109 (58.9)	1,328 (53.8)
Female	201 (61.3)	162 (63.8)	775 (41.1)	1,138 (46.2)
Race				
Caucasian	235 (77.1)	168 (74.3)	1,208 (73.0)	1,611 (73.7)
African American	31 (10.2)	30 (13.3)	170 (10.3)	231 (10.6)
Asian/Pacific Islander	4 (1.3)	1 (0.4)	24 (1.5)	29 (1.3)
Hispanic	29 (9.5)	20 (8.9)	200 (12.1)	249 (11.4)
Native American	1 (0.3)	2 (0.9)	7 (0.4)	10 (0.5)
Other	5 (1.6)	5 (2.2)	46 (2.7)	56 (2.5)
Survival				
Survived	321 (92.8)	247 (96.9)	1,774 (94.1)	2,342 (94.2)
Died	25 (7.2)	8 (3.1)	112 (5.9)	145 (5.8)
Severe complication				
Yes	32 (9.3)	21 (8.2)	209 (11.1)	262 (10.5)
No	314 (90.8)	234 (91.8)	1,677 (88.9)	2,225 (89.5)
Fracture location				
Ilium	41 (11.9)	47 (18.4)	228 (12.1)	316 (12.7)
Ischium	7 (2.0)	7 (2.8)	53 (2.8)	67 (2.7)
Pubis	222 (64.2)	143 (56.1)	1,059 (56.2)	1,424 (57.3)
Multiple	31 (9.0)	30 (11.8)	277 (14.7)	338 (13.6)
Unspecified	10 (2.9)	4 (1.6)	70 (3.7)	84 (3.4)
Other	35 (10.0)	24 (9.3)	199 (10.5)	258 (10.3)
Systolic blood pressure (mmHg)				
<90	55 (15.9)	17 (6.7)	176 (9.3)	248 (10.0)
90–139	173 (50.0)	178 (69.8)	1,197 (63.5)	1,548 (62.2)
140+	118 (34.1)	60 (23.5)	513 (27.2)	691 (27.8)
MOI				
Motor vehicle	127 (36.7)	228 (89.4)	1,094 (58.0)	1,449 (58.3)
Motorcycle	17 (4.9)	6 (2.4)	196 (10.4)	219 (8.8)
Pedestrian	17 (4.9)	5 (2.0)	70 (3.7)	92 (3.7)
Crush	13 (3.8)	5 (2.0)	101 (5.4)	119 (4.8)
High fall	52 (15.0)	4 (1.5)	248 (13.2)	304 (12.2)
Low fall	120 (34.7)	7 (2.7)	177 (9.3)	304 (12.2)
AIS of pelvis				
3	321 (92.7)	231 (90.6)	1,606 (85.2)	2,158 (86.8)
4	20 (5.8)	17 (6.6)	225 (11.9)	262 (10.5)
5	5 (1.5)	7 (2.8)	55 (2.9)	67 (2.7)
ISS				
<25	284 (82.1)	169 (66.3)	1,308 (69.4)	1,761 (70.8)
≥25	62 (17.9)	86 (33.7)	578 (30.6)	726 (29.2)
Pneumonia				
Yes	3 (0.9)	12 (4.7)	53 (2.8)	68 (2.7)
No	343 (99.1)	243 (95.3)	1,833 (97.2)	2,419 (97.3)
Acute respiratory stress syndrome				
Yes	1 (0.3)	1 (0.4)	28 (1.5)	30 (1.2)
No	345 (99.7)	254 (99.6)	1,858 (98.5)	2,457 (98.8)
Deep vein thrombosis				
Yes	0 (0.0)	1 (0.4)	19 (1.0)	20 (0.8)
No	346 (100.0)	254 (99.6)	1,867 (99.0)	2,467 (99.2)
Bacteremia				
Yes	1 (0.3)	1 (0.4)	6 (0.3)	8 (0.3)
No	345 (99.7)	254 (99.6)	1,880 (99.7)	2,479 (99.7)
Renal failure				
Yes	2 (0.6)	1 (0.4)	7 (0.4)	10 (0.4)
No	344 (99.4)	254 (99.6)	1,879 (99.6)	2,477 (99.6)
Pulmonary embolism				
Yes	1 (0.3)	0 (0.0)	7 (0.4)	8 (0.3)
No	345 (99.7)	255 (100.0)	1,879 (99.6)	2,479 (99.7)
ICU stay				
<1 week	326 (94.2)	224 (87.8)	1,676 (88.9)	2,226 (89.5)
1 week	20 (5.8)	31 (12.2)	210 (11.1)	261 (10.5)
Hospital stay				
<1 week	256 (74.0)	151 (59.2)	1,011 (53.6)	1,418 (57.0)
1 week	90 (26.0)	104 (40.8)	875 (46.4)	1,069 (43.0)
Major procedure				
CRIF	0 (0.0)	0 (0.0)	1 (0.1)	1 (0.1)
ORIF	31 (9.0)	45 (17.6)	501 (26.5)	577 (23,1)
Closed dislocation reduction	0 (0.0)	2 (0.8)	7 (0.4)	9 (0.4)
Open dislocation reduction	0 (0.0)	0 (0.0)	13 (0.7)	13 (0.5)
No major procedure	315 (91.0)	208 (81.6)	1,364 (72.3)	1,887 (75.9)

*MOI* mechanism of injury, *AIS* Abbreviated Injury Scale, *ISS* Injury Severity Score, *ICU* intensive care unit, *CRIF* closed reduction internal fixation, *ORIF* open reduction internal fixation

### Bivariate analysis for the severe pelvic fracture subpopulation

Similar to what was done with the whole population, bivariate analysis was also performed on the severe pelvic fracture subpopulation (Table 5). As compared to the adults, children had higher odds of death but lower odds of severe complication. However, adolescents had a crude decrease in odds of death and severe complication after sustaining a pelvic fracture compared to the adults. Individuals suffering from hypovolemic shock, head injury, and ISS ≥25 had an increase in odds of death and severe complications. Motor vehicle accidents caused the largest increase in odds of death (OR 1.58, 95 % CI 1.10–2.26), while motorcycle accidents accounted for the largest increase in severe complication (OR 1.58, 95 % CI 1.06–2.34).

**Table 5:** Bivariate analysis for isolated closed pelvic fractures with an associated AIS ≥3, comparing predictors upon arrival to the emergency department with the outcomes death and severe complication (odds ratios and 95 % confidence intervals)

Predictors	Outcome	
	Death, *n* = 2,487	Severe complication^a^, *n* = 2,487
Male	1.11 (0.79–1.56)	**1**.**60** (**1**.**22**–**2**.**08**)
Not Caucasian	**1**.**53** (**1**.**09**–**2**.**15**)	1.26 (0.97–1.64)
Pediatric (<18 years)	0.92 (0.62–1.37)	0.78 (0.57–1.06)
Children (<13 years)	1.31 (0.84–2.05)	0.85 (0.57–1.25)
Adolescent (13–17 years)	0.50 (0.24–1.02)	0.74 (0.47–1.18)
Adults (18–54 years)	1.09 (0.73–1.62)	1.29 (0.94–1.77)
Hypovolemic shock	**13**.**0** (**9**.**06**–**18**.**6**)	**7**.**35** (**5**.**44**–**9**.**93**)
Head injury	**4**.**41** (**2**.**94**–**6**.**60**)	**5**.**91** (**4**.**28**–**8**.**16**)
ISS ≥25	**27**.**5** (**9**.**92**–**12**.**5**)	**14**.**9** (**10**.**7**–**20**.**7**)
Motor vehicle accident	**1**.**58** (**1**.**10**–**2**.**26**)	**1**.**32** (**1**.**01**–**1**.**72**)
Motorcycle accident	1.62 (0.98–2.67)	**1**.**58** (**1**.**06**–**2**.**34**)
Fall from height	0.63 (0.34–1.16)	0.74 (0.48–1.13)
Low-energy fall	**0**.**095** (**0**.**023**–**0**.**38**)	**0**.**35** (**0**.**20**–**0**.**61**)
Crush	0.85 (0.37–1.97)	0.95 (0.52–1.75)
Pedestrian versus auto	1.33 (0.60–2.93)	1.33 (0.60–2.93)

Bold values signify 95 % confidence intervals that are completely greater than 1, indicating a significant positive association

*ISS* Injury Severity Score

^a^Severe complication is defined as having one or more of the following during hospital course: pneumonia, bacteremia, deep vein thrombosis, pulmonary embolism, renal failure, acute respiratory distress syndrome, or death

### Multivariate analysis for the severe pelvic fracture subpopulation

Variables that were found to be significant in the bivariate analysis were included in the final multiple logistic regression models for death and severe complication as outcomes (Table 6). When controlling for mechanism of injury and other prehospital conditions and using the adult group as a referent, children had nearly double the odds of death after sustaining a severe pelvic fracture, whereas adolescents had a large decrease in odds of death (OR 0.40, 95 % CI 0.18–0.91). Compared to adults, children had potentially increased odds of severe complication after sustaining a severe pelvic fracture (OR 1.07, 95 % CI 0.65–1.75), whereas adolescents had potentially decreased odds of severe complication (OR 0.66, 95 % CI 0.39–1.14) following a severe pelvic fracture.

**Table 6:** Multivariate logistic regression analysis for isolated closed pelvic fractures with an associated AIS ≥3, using predictors upon arrival to the emergency department with the outcomes death and severe complication (odds ratios and 95 % confidence intervals)

Model predictors	Outcome	
	Death, *n* = 2,487	Severe complication^a^, *n* = 2,487
Children (<13 years)	**1**.**90** (**1**.**06**–**3**.**41**)	1.07 (0.65–1.75)
Adolescent (13–17 years)	**0**.**40** (**0**.**18**–**0**.**91**)	0.66 (0.39–1.14)
Adults (18–54 years)	Referent	Referent
Male	–	1.31 (0.95–1.80)
Not Caucasian	1.34 (0.90–2.01)	–
Hypovolemic shock	**9**.**93** (**6**.**52**–**15**.**1**)	**6**.**56** (**4**.**57**–**9**.**42**)
Head injury	**1**.**70** (**1**.**05**–**2**.**77**)	**2**.**51** (**1**.**72**–**3**.**66**)
ISS ≥25	**19**.**3** (**10**.**5**–**35**.**5**)	**10**.**5** (**7**.**23**–**15**.**2**)
Motor vehicle accident	0.96 (0.62–1.50)	0.98 (0.65–1.49)
Motorcycle accident	–	1.27 (0.72–2.23)
Low-energy fall	0.25 (0.06–1.12)	0.96 (0.47–1.99)
Hosmer–Lemeshow^b^	0.29	0.14

Bold values signify 95 % confidence intervals that are completely greater than 1, indicating a significant positive association

*ISS* Injury Severity Score

^a^Severe complication is defined as having one or more of the following during hospital course: pneumonia, bacteremia, deep vein thrombosis, pulmonary embolism, renal failure, acute respiratory distress syndrome, or death

^b^Hosmer–Lemeshow *p*-value given for model’s goodness of fit instead of odds ratio and 95 % confidence interval

In this model, an ISS >25, hypovolemic shock, and head injury yielded a substantial increase in odds of death (Table 6).

## Discussion

In this study, we analyzed data gathered from the NTDB to characterize the risks for mortality and complications associated with pelvic fractures in adolescents as compared to younger children and adults. Compared to adults, adolescents were shown to have decreased mortality and severe complication rates, while children have an increased mortality rate but decreased severe complication rate. In the subanalysis of severe pelvic fractures, the same pattern emerged, although the results for complication rates were not statistically significant.

Previous studies have shown conflicting reports regarding mortality associated with pediatric pelvic fractures. Mortality rates as low as 1.4 % to as high as 25 % have been reported [5, 10, 11, 14, 15]. Banerjee et al. [7] reported a 16 % mortality rate in 44 patients related mainly to the associated injuries and not to the pelvic fractures themselves. Another study reported 5 % overall mortality for 722 pediatric pelvic fractures versus 17 % among similar injuries in the adult population [15]. Comparable to previously reported studies, the current study reported 10.2 % survival for children and only 6.8 % for adolescents, compared to 8.5 % in adults with pelvic fractures. The pattern of lower survival in children and higher survival in adolescents compared to adults was also seen in the analysis of severe pelvic injuries (Table 4). This study illustrates that simply comparing pediatric mortality to that of adults may be an inadequate evaluation due to the fact that adolescents and children sustaining pelvic injuries may have different outcomes.

Central nervous system head injury was cited as the most common cause of death in children with pelvic fractures in two recent studies [2, 11]. Other causes of death in children with pelvic fractures, reported in these studies, include multiorgan failure and visceral injuries [2, 11]. In a study that examined the National Pediatric Trauma Registry (NPTR), hospital type (children’s or general) and ISS were the only significant parameters in the multivariate analysis [8]. Hemorrhage from a pelvis fracture-related vascular injury was reported as the cause of death in only 0.3 % of children, compared with 3.4 % of adults [15]. The current study supports these previous studies. Multivariate analysis showed hypovolemic shock, head injury, and ISS ≥25 to correlate with an increased mortality rate in a patient with any type of pelvic fracture (severe or not). The multivariate model for severe pelvic injuries also demonstrated that, despite their lower ISS, children had a significantly higher risk of mortality (OR 1.90, 95 % CI 1.06–3.41) and adolescents had a significantly lower risk of mortality (OR 0.40, 95 % CI 0.18–0.91) as compared to adults.

Whereas many studies report the rate of associated injuries, very few studies report the rate of complications after pediatric pelvic fractures [2]. According to a previous study of 120 children (age <16 years) with pelvic fractures, complications included 13.3 % wound complications, 8.3 % urinary tract infections, 4.2 % septicemias, 1.7 % ARDS, and one case (0.8 %) of pulmonary embolus (PE) [2]. In the current study, there were 346 children (age <13 years) with severe pelvic injuries, with a lower overall complication rate of 9.3 %. There was one case (0.3 %) of ARDS and one case of PE (0.3 %), two cases of renal failure (0.6 %), and no cases of septicemia. The 255 adolescents with severe pelvic injury had an even lower complications rate of 8.2 %, with one ARDS (0.4 %), one septicemia (0.4 %), one renal failure (0.4 %), one DVT, but no PE. Urinary tract infection (UTI) was not considered to be a significant complication in our study. However, in the severe pelvic injury group, there were three children (0.87 %), three adolescents (1.2 %), and 30 adults (1.59 %) with UTI. The most common severe complication was pneumonia: three children (0.87 %), 12 adolescents (4.7 %), and 53 adults (2.8 %).

Previous studies have reported a high percentage of associated injuries in the pediatric pelvic fracture population. In one study surveying 166 cases, there were 38.6 % head traumas, 19.9 % significant chest traumas, and 19.3 % abdominal/visceral trauma [11]. Additionally, an incidence of 11–27 % of associated abdominal injuries [2, 10, 11, 15–17], 27 % associated chest injuries, and up to 50 % of associated head injuries have been previously reported [2, 10, 18]. The proportion of associated injuries (head, thorax, and abdomen ,respectively) in the current study was 7.5, 4.1, and 4.6 % for children; 18, 7.1, and 8.6 % for adolescents. The proportion of a depressed consciousness level [Glasgow Coma Scale (GCS) score < 15] in the population of severe pelvic fractures was 19.4 % in children and 31.3 % in adolescents (25 % if adolescents and children are grouped together). This is different to the previously reported 38 % in the pediatric population [2].

Other pertinent findings in this study included a 5–7 times higher incidence of pelvic fractures in adults versus children and adolescents, a higher incidence of pelvic fractures in white non-Hispanic males, the vast majority of the injury mechanism of pelvic fractures being blunt trauma from motor vehicle accidents, and a predominance of non-operative treatment for these fractures. These findings are largely in accordance with previous reports [2, 3, 8, 11].

The main strengths of the current study is that this is the largest cohort used for an investigation of this sort, and the national representation of academic and non-academic centers of various levels provide far-reaching generalizability. However, this study also has a number of limitations. While the NTDB includes a very large sample of trauma centers from all over the United States, it is a convenience sample that consists solely of data submitted by participating hospitals, leading to a disproportionate number of larger hospitals with younger and more severely injured patients. The quality of data entered into the NTDB is solely dependent on the participants entering the data; however, the NTDB is continuously working to screen, clean, and standardize the quality of the data entered into the database [13]. The larger population provided by the NTDB may be the reason for a higher mortality seen in our pediatric population, unlike the previous studies with smaller sample sizes. The NTDB also reported more pelvic fractures in children than in adolescents, which contributed to our pediatric population’s higher mortality rate. We also used pelvis AIS ≥3 to indicate a severe pelvic injury, while other studies have used a cutoff of AIS ≥4 [3]. Additionally, since the NTDB does not code separately for pelvic AIS, we derived our pelvic AIS score by using the lower extremity AIS score and excluding all patients with other lower extremity injuries. Assuming an associated lower extremity fracture rate of about 25 % [11], this may have influenced our descriptive statistics. However, this allowed us to compare similar injuries and better isolate the effect of age. The NTDB also does not provide blood gas values such as base deficit and lactate, and, therefore, our analysis was limited to the use of hypovolemic shock on arrival. There were also weaknesses in the tests of the model fitness. This suggests that there could be interactions between variables that were not included in the model. These variables might not have been collected by the NTDB or may be latent factors that are not identified. Such discrepancies may have altered our final parameter estimates by either inflating or deflating their values. Finally, we chose the age group of 13–17 years to represent adolescence. Biological, as well as social and environmental, changes influence the beginning of adolescence, making the precise beginning age questionable. Because of this, our age group was chosen based on previous publications [1, 12].

In conclusion, the results of this study suggest that the adolescents with pelvic fractures behave as a separate group from the children and adult populations. These results emphasize the need for further epidemiological research on disparate injury response between pediatric subpopulations defined by level of maturity. There should be further research aimed towards designating physiological criteria besides age by which to place pediatric patients in their respective subpopulations. This study highlights the fact that the mortality rate seen in a given pediatric population may not be an accurate measurement, as our lower mortality rate largely stems from the adolescents’ increased survival, while the children may actually have a higher mortality rate than the adults.
